# Adipose tissue-derived small extracellular vesicles modulate macrophages to improve the homing of adipocyte precursors and endothelial cells in adipose tissue regeneration

**DOI:** 10.3389/fcell.2022.1075233

**Published:** 2022-12-06

**Authors:** Jia Dong, Bin Wu, Weidong Tian

**Affiliations:** ^1^ Department of Stomatology, The People’s Hospital of Longhua Shenzhen, Shenzhen, China; ^2^ State Key Laboratory of Oral Disease, National Engineering Laboratory for Oral Regenerative Medicine, National Clinical Research Center for Oral Diseases, West China School of Stomatology, Sichuan University, Chengdu, China; ^3^ Department of Oral and Maxillofacial Surgery, West China Hospital of Stomatology, Sichuan University, Chengdu, China

**Keywords:** adipose tissue-derived small extracellular vesicles, macrophages, adipocyte precursors, endothelial cells, tissue regeneration

## Abstract

Rapid infiltration of endogenous cells induced by cell-free biomaterials is the first and crucial step in tissue regeneration and macrophage is largely involved. Our previous study reported adipose tissue-derived small extracellular vesicles (sEV-AT) could successfully promote adipose tissue regeneration. However, the role of macrophages in this process was unknown. In this study, we isolated sEV-AT and subcutaneously implanted it into the back of SD rats. The results showed sEV-AT increased macrophage infiltration significantly, which was followed by improving homing of adipocyte precursors (APs) and endothelial cells (ECs). However, when macrophages were depleted by clodronate liposome within 1 week, the homing of APs and ECs, and adipose tissue regeneration were destroyed. *In vitro*, sEV-AT showed the ability to promote the migration of macrophages directly. Besides, sEV-AT-pretreated macrophages improved the migration of APs and ECs, accompanied by the increase of chemokines (MCP-1, SDF-1, VEGF, and FGF) and the activation of NF-kB signaling pathway. These findings indicated sEV-AT might regulate the secretion of chemokines *via* activating NF-kB signaling pathway to improve homing of APs and ECs and facilitate adipose tissue regeneration. These findings deepened our understanding of small extracellular vesicle-induced tissue regeneration and laid a theoretical foundation for the clinical application of sEV-AT.

## Introduction

It was known that all implants would evoke the host’s immune response. Previously, the host’s immune response was considered to be harmful, which was needed to avoid or blunt. In recent years, it became versatile and even beneficial to tissue repair (Whitaker et al., 2021). The host’s immune response to implants was largely commanded by macrophages, one of the most abundant immune cells. Both resident tissue macrophages and monocytes/macrophages in blood displayed multiple functions to clear debris and secret cytokines and chemokines, which modulated inflammation and recruited other cells to promote tissue regeneration (Koh and DiPietro, 2011; O'Brien et al., 2019). On the contrary, the depletion of macrophages after tissue injury would lead to the impairment of tissue regeneration (Tidball and Wehling-Henricks, 2007). In addition, the study of macrophages in adipose inflammation and obesity also suggested the important role of macrophages in adipose development and regeneration.

Previous studies mainly focused on the phenotype-switching and the inflammatory regulation of macrophages under biomaterial stimulation (Novak and Koh, 2013; Jetten et al., 2014; Jetten et al., 2014; Spiller et al., 2014; Gurevich et al., 2018; Deng et al., 2019). However, macrophages not only played a pro-inflammatory or anti-inflammatory role by secreting inflammatory factors but also guided cellular migration and promoted the cell’s differentiation by secreting a variety of chemokines (like SDF-1, MCP-1 VEGF, and FGF) (Arango Duque and Descoteaux, 2014). Up to now, there are few systematic and comprehensive studies on the ability of macrophage chemoattraction, which might deepen our understanding of the roles of macrophages in tissue regeneration.

How to repair soft tissue defects caused by congenital malformations, trauma and cancer was still a challenge in the field of plastic surgery. Adipose tissue engineering was considered to be a promising method for soft tissue replacement (Choi et al., 2010). Small extracellular vesicles (sEVs), released from different cells and tissues, carried multiple proteins, nucleic acids, and lipids (Théry et al., 2018; Flaherty et al., 2019; Jeppesen et al., 2019; Raposo and Stahl, 2019; Yang et al., 2019). For tissue regeneration, sEVs have been reported to recruit host cells, regulate immune responses, stimulate angiogenesis, and promote tissue repair or regeneration (Qi et al., 2016; Zhang et al., 2016; Ahn et al., 2018). Our previous study has shown that adipose tissue-derived small extracellular vesicles (sEV-AT), possessed the typical characteristics of sEVs, could effectively promote adipose tissue regeneration (Dai et al., 2017; Dong et al., 2020; He et al., 2022). However, the interaction between sEV-AT and the host’s immune system, especially macrophages, was unknown. Moreover, the role of macrophages in this regeneration process was exclusive.

In this study, we first implanted sEV-AT *in vivo*. The infiltration of macrophages and the following homing of adipocyte precursors (APs) and endothelial cells (ECs) induced by sEV-AT were evaluated and compared with the blank group. Besides, we also depleted macrophages at the early stage of sEV-AT implantation by clodronate liposome injection. Then, macrophages, APs, ECs, and adipose tissue regeneration in the clodronate liposome (CL) group were compared with that of sEV-AT group to confirm the function of macrophages. *In vitro*, we co-cultured sEV-AT-pretreated macrophages with APs or ECs to investigate the chemoattraction ability of macrophages induced by sEV-AT. Finally, the concentration of chemokines (MCP-1, SDF-1 VEGF, and FGF) and the activation of NF-kB signaling pathway in macrophages were evaluated.

## Materials and methods

### Animals

Sprague Dawley (SD) rats were obtained from Chengdu Dashuo experimental animal Co., Ltd. (China). All operations of animals were reviewed and approved by the Ethics Committees of the State Key Laboratory of Oral Diseases, West China School of Stomatology, Sichuan University (approval number: WCHSIRB-D-2020-391).

### sEV-AT isolation

sEV-AT was isolated from the inguinal adipose tissue of 4-week-old SD rats. Briefly, adipose tissue was cut into small pieces and transferred into a flask to culture for 2 days. Then, the supernatant was collected, filtered, and concentrated. The concentrated extract medium was mixed with 0.5 volume of Total Exosome Isolation^TM^ reagent (Life Technologies, United States), incubated overnight at 4°C, and spun down at 10,000 g, 4°C for 1 h. The obtained pellet was resuspended in PBS for further testing. More details were shown in our previous study (Dong et al., 2020).

### Analysis of sEV-AT

sEV-AT were fixed, negative stained, and imaged by transmission electron microscope (TEM, Tecnai G2 F20 S-TWIN, United States). The size distribution was measured by ZetaVIEW S/N 19–480 analysis system (Germany). The protein markers (HSP70, CD81, TSG101, and actin) were detected by western blot. For cellular uptake, sEV-AT were labeled with DiO (Invitrogen) in RPMI-1640 medium (Hyclone) at 37°C for 20 min. Then, DiO-labeled sEV-AT were collected by the Total Exosome Isolation^TM^ reagent and cultured with Raw 264.7 cells for 6 h. Then, these cells were fixed by paraformaldehyde, stained by DAPI and phalloidin (Invitrogen), and imaged by confocal microscopy (FV1000, Olympus, Japan). More details were shown in our previous study (Dong et al., 2020).

### Animal experiments

4-week-old SD rats (n = 45) were divided into 3 groups: the blank group (n = 15), the sEV-AT group (n = 15), and the clodronate liposome injected (CL) group (n = 15). In the blank group, 120 μL Matrigel was put into a custom-designed tube to maintain the integrity and shape, and the tube was subcutaneously implanted into the back of SD rat. In the sEV-AT group, 120 μL Matrigel containing 360 μg sEV-AT was also put into the custom-designed tube and was subcutaneously implanted. More details of the implantation operation were shown in our previous study (Dong et al., 2020). In the CL group, 0.1 ml of the suspension of clodronate liposome (LIPOSOME, Netherlands) per 10 g animal weight was intravenously injected into each rat. After 2 days of injection, the same implantation as the sEV-AT group was performed. The clodronate liposome was injected into the rats of the CL group every 2 days within 1 week to maintain the depletion of macrophages. Rats were sacrificed at 3 days, 5 days, 1 week, 2 weeks, and 4 weeks (n = 3 per timepoint per group), and the liver, the spleen, and the implant were isolated for further testing.

### Histological analysis

The implants, livers, and spleens were isolated at 3 days, 5 days, 1 week, 2 weeks, and 4 weeks, fixed with 4% paraformaldehyde, dehydrated with graded ethanol, and embedded with paraffin. The embedded implants were sectioned into 5–6 µm sections. Hematoxylin and eosin (H&E, Solarbio, China) staining was carried out. For immunohistochemical staining, the sections were blocked for 30 min and incubated with primary antibodies against F4/80 (Abcam) or CD31 (Novus Bio) overnight at 4°C. The DAB kit (Gene Tech, Shanghai, China) was used for showing the secondary antibody. For immunofluorescence staining, the sections were also blocked for 30 min and incubated with the mixed primary antibodies against CD29 (Abcam) and CD34 (Abcam) overnight at 4°C, followed by secondary antibodies goat anti-mouse 488 (Invitrogen) and chicken anti-rabbit 555 (Invitrogen). The nucleus was stained with DAPI. Images were captured by confocal microscopy (FV1000, Olympus, Japan).

### Cell culture

Macrophages (Raw264.7 cell line) were cultured in RPMI-1640 medium (HyClone), 10% inactivated fetal bovine serum (FBS, Gibco). Adipocyte precursors (APs, 3T3-L1 cell line) were cultured in a-MEM medium (HyClone), 10% fetal bovine serum (FBS, Gibco). Endothelial cells (ECs) were isolated from the thoracic aorta of SD rats according to the previous method reported by Tian et al. (2014) and were cultured in endothelial cells growth medium (EGM- 2MV; Lonza).

### Scratch assays

1 × 10^5^ macrophages were seeded in a 24-well plate and pipette tips were used for scratching according to the marked line. Then, the cells were divided into 3 groups (n = 3 per group): the blank group, the 50 ug/ml sEV-AT group, and the 100 ug/ml sEV-AT group. The cells in the blank group were cultured with 500 μL culture medium only. The cells in other groups were cultured with 500 μL culture medium containing 50 or 100 ug/ml sEV-AT, respectively. Images were captured at 0 and 30 h and the migrated area was measured by Image Pro Plus software.

### Transwell assays

1 × 10^5^ macrophages were seeded in a 24-well plate. Then, the cells were divided into 3 groups (n = 3 per group): the blank group, the 50 ug/ml sEV-AT group, and the 100 ug/ml sEV-AT group. The cells in the blank group were cultured with 500 μL culture medium only. The cells in other groups were cultured with 500 μL culture medium containing 50 or 100 ug/ml sEV-AT, respectively. After culturing for 6 h, the culture medium was removed. At the same time, 1.5 × 10^4^ APs or ECs were seeded onto the upper layer to set up two co-culture systems. After co-culturing for 12 h, non-migrated cells were removed by cotton swab, and migrated cells were fixed with 4% paraformaldehyde and stained with 0.1% crystal violet (Sigma, United States) (n = 3 per group). Images were captured and the number of migrated cells was measured by Image Pro Plus software.

### ELISA

The culture medium, in the above co-culture system, was collected, and the debris of cells was removed by centrifugation at 3,000 rpm for 10 min. Supernatants were analyzed by MCP-1 ELISA kit (DECO), SDF-1 ELISA kit (DECO), VEGF ELISA kit (DECO), and FGF ELISA kit (DECO), according to the manufacturer’s protocols.

### Western blot

The macrophages, in the above co-culture system, were collected. RIPA Lysis Buffer (KeyGEN) was used as a dissolving reagent. 15% polyacrylamide gel was used for resolving the proteins and a nitrocellulose membrane was used for blotting. The nitrocellulose membranes were blocked followed by incubating with primary antibodies, p50 (Abcam), p65 (Cell Signaling Technology), phosphorylated-p50 (Ser927) (NovoPro), phosphorylated-p65 (Ser536) (Cell Signaling Technology), and Actin (Abcam), overnight at 4°C, Horseradish peroxidase (HRP)-conjugated secondary antibodies were inhibited for 4 h at 20–30°C. High-sig ECL Western Blotting Substrate (Tanon) was used for detecting the signals and ImageQuant LAS 4000 mini machine (GE Healthcare) was used for capturing the images.

### Statistical analysis

All statistical analyses were performed using Microsoft Excel or GraphPad Prism 7 software. For statistical analysis of values, all results represent the mean ± standard deviation of three samples. For the statistical analysis of pictures, the mean of three visual fields is taken for each sample, and then the mean ± standard deviation was further used for analyzing three samples. Ordinary one-way ANOVA followed by Tukey’s multiple comparisons test was used for determining the level of significance. *p < 0.05, **p < 0.01, ***p < 0.001, ****p < 0.0001.

## Results

### Characterization of sEV-AT and cellular uptake

**FIGURE 1:**
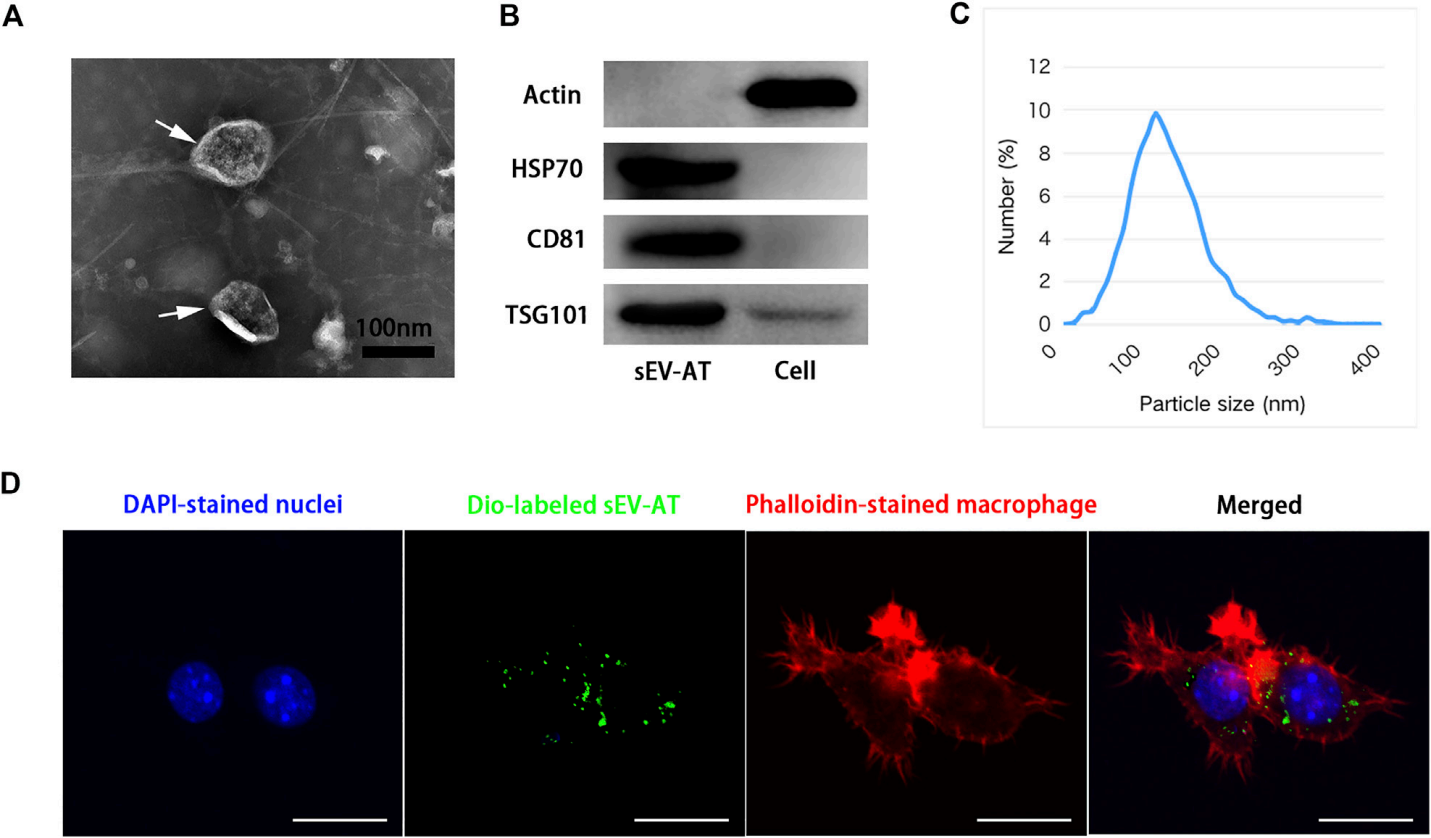
Characterization of sEV-AT. **(A)** Representative images of sEV-AT with transmission electron microscopy. The white arrows pointed out the sEV-AT. Scale bar = 100 nm. **(B)** Western blot analysis of exosomal makers, HSP70, CD81, and TSG101. Actin was cellular a protein as a control. **(C)** The particle size distribution of sEV-AT was measured by ZataView analysis. **(D)** Uptake analysis of sEV-AT by macrophages (blue: DAPI-stained nuclei; green: Dio-labeled sEV-AT; red: phalloidin-stained macrophage). Scale bar = 10 µm.

sEV-AT were isolated from rat adipose tissue according to our previous study (Dong et al., 2020). sEV-AT were round-shaped vesicles surrounded by a bilayer membrane, which were analyzed by Transmission electron microscopy (TEM) (Figure 1A). High presence of exosome-enriched protein markers (HSP70, CD81, and TSG101) was detected in sEV-AT by western blot analysis, and the cellular protein marker, actin, was detected in cells but not in sEV-AT (Figure 1B). The size and distribution of sEV-AT were measured and the result showed that sEV-AT was peaked at about 120 nm (Figure 1C). Besides, we confirmed the Dio-labeled sEV-AT could be taken into the macrophages after 6 h of culturing (Figure 1D).

### sEV-AT promoted the infiltration of macrophages *in vivo*

**FIGURE 2:**
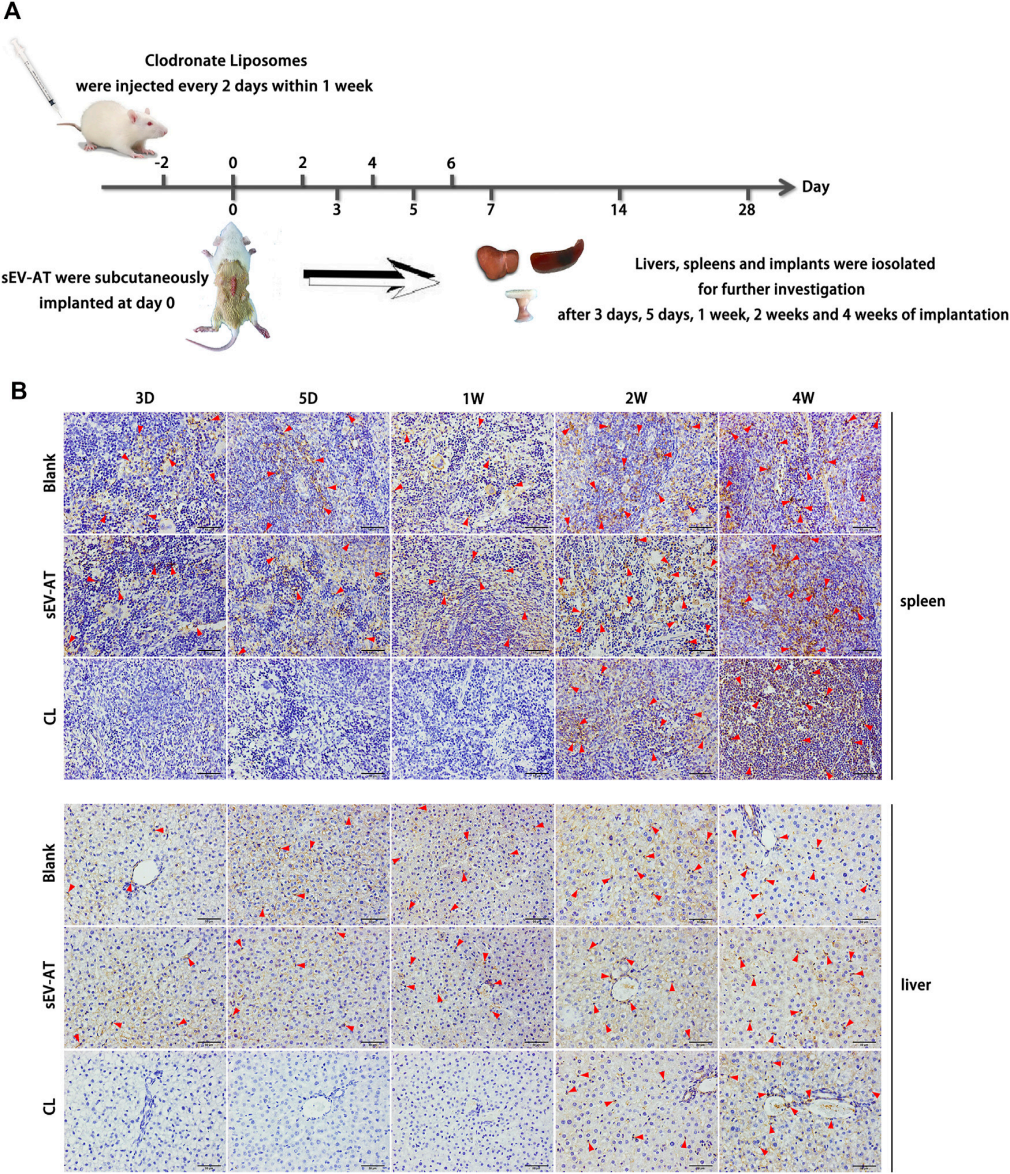
Macrophages in the livers and spleens. **(A)** Clodronate liposome was intravenously injected into each rat in the CL group. After 2 days of injection, sEV-AT mixed with Matrigel was subcutaneously implanted. Clodronate liposome was injected into the rats of the CL group every 2 days within 1 week to maintain the depletion of macrophages. The liver, the spleen, and the implant were isolated for further testing at 3 days, 5 days, 1 week, 2 weeks, and 4 weeks. **(B)** Representative anti-F4/80 immunohistochemical images of the livers and spleens. The red triangles pointed out typical F4/80^+^ cells. Scale bar = 50 µm.

To investigate the effect of sEV-AT on macrophage infiltration and further study the effect of macrophage depletion on adipose tissue regeneration induced by sEV-AT *in vivo*, all animals were divided into 3 groups: the blank group, the sEV-AT group, and the clodronate liposome (CL) group. Clodronate liposome was intravenously injected into each rat in the CL group. After 2 days of injection, sEV-AT mixed with Matrigel was implanted into the back of SD rats in the sEV-AT group and the CL group. The blank group was implanted with Matrigel only. The clodronate liposome was injected into the CL group every 2 days within 1 week to maintain the depletion of macrophages. After 3 days, 5 days, 1 week, 2 weeks, and 4 weeks of implantation, the implants, the livers, and the spleens were isolated for further investigation (Figure 2A). The pan macrophage marker F4/80 was used for quantifying macrophages and the results indicated the injection of clodronate liposome every 2 days could continuously deplete of macrophages based on the results that there was almost no F4/80^+^ cells in livers and spleens in the CL group at 3 days, 5 days, and 1 week. However, when clodronate liposome injection was stopped, F4/80^+^ macrophages reappeared in the livers and spleens, indicating clodronate liposome did not damage the function of macrophage production in rats (Figure 2B).

**FIGURE 3:**
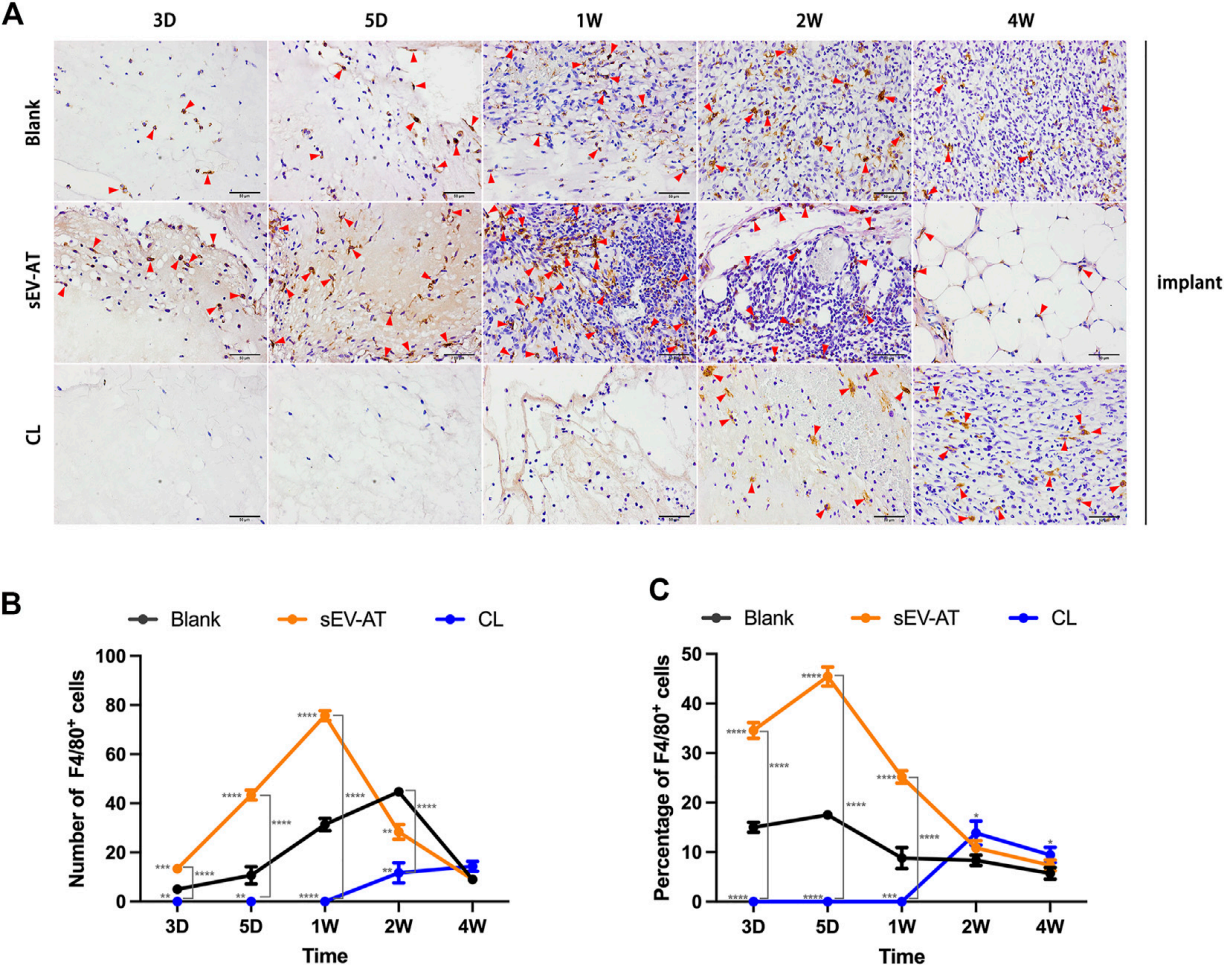
Infiltrated macrophages in the implants. **(A)** Representative anti-F4/80 immunohistochemical images of the implants. The red triangles pointed out typical F4/80^+^ cells. Scale bar = 50 µm. **(B)** The number of F4/80^+^ macrophages per field of view (scar bar = 50 μm) was analyzed (n = 3). **(C)** The percentage of F4/80^+^ macrophages in total cells per field of view (scale bar = 50 µm) was analyzed (n = 3). The significance was tested with ordinary one-way ANOVA followed by Tukey’s multiple comparisons test. (**/***/**** marked beside the data point represented the comparison between the sEV-AT group/the CL group and the blank group, **p < 0.01, ***p < 0.001, ****p < 0.001).

Immunohistochemical staining of the implants showed sEV-AT significantly promoted F4/80^+^ macrophage infiltration in the implants at the early stage (Figure 3A) compared with the blank group. In the sEV-AT group, the number of macrophages in each field was about twice that of the blank group at 3 days, 5 days, and 1 week. The largest number of macrophages appeared at 1 week. For the percentage of macrophages, sEV-AT also increased it to about twice that of the blank group at 3 days, 5 days, and 1 week (Figure 3B). However, the largest percentage of macrophages appeared at 5 days in the sEV-AT group (Figure 3C). The decrease in the percentage of macrophages at 1 week might be related to the increase in the number of other functional cells (such as adipocyte precursors and endothelial cells). Besides, the injection of clodronate liposome every 2 days could successfully eliminate the infiltration of macrophages into the implants based on the results that there were no F4/80^+^ cells in the implants within 1 week (Figure 3).

These results suggested sEV-AT could significantly increase and accelerate the recruitment of macrophages into implants and clodronate liposome injection at the early stage of implantation could successfully inhibit this function.

### Early recruitment of macrophages promoted the homing of APs and ECs

**FIGURE 4:**
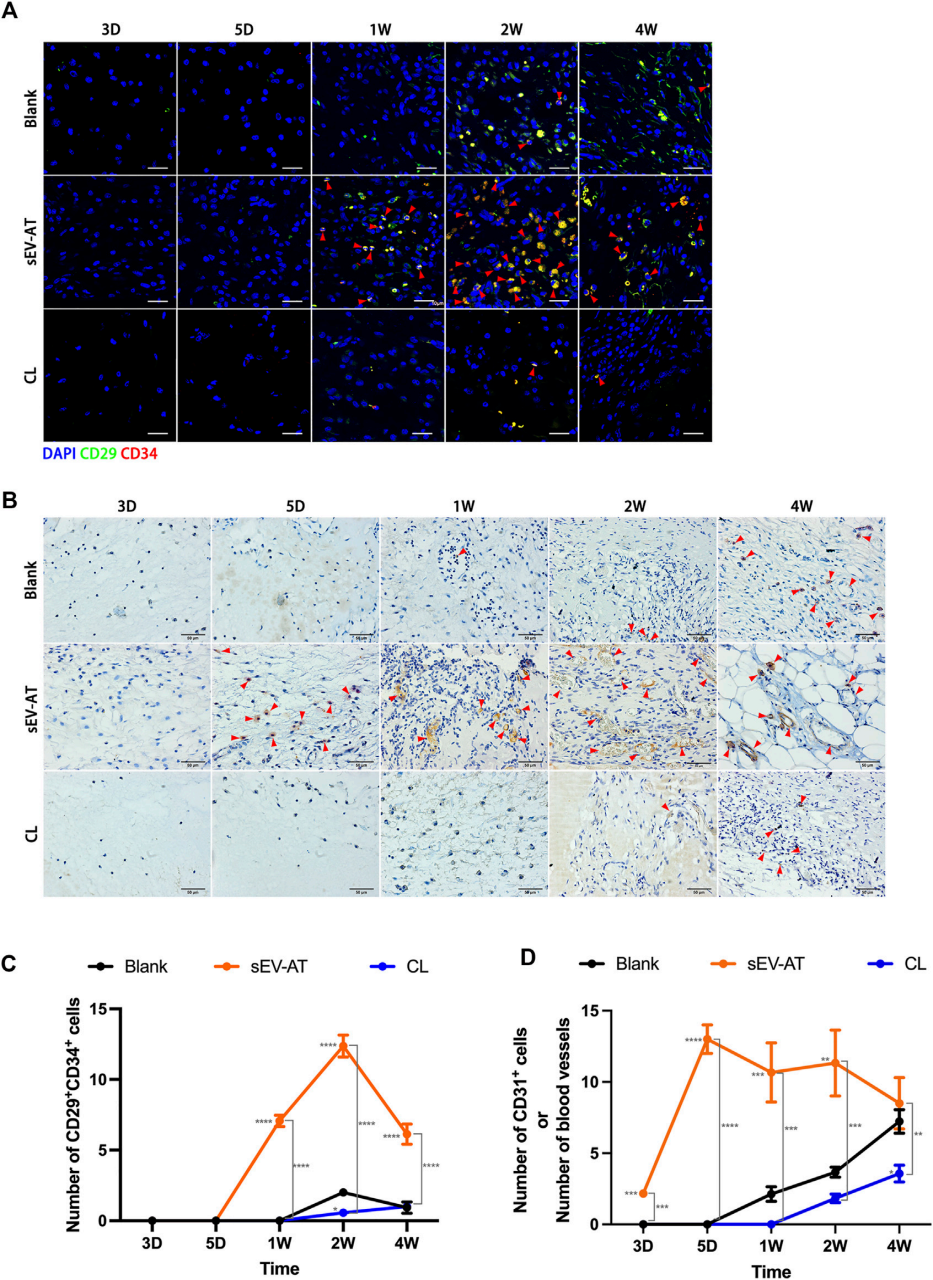
The homing of APs and ECs in the implants. **(A)** Representative anti-CD29 and anti-CD34 Immunofluorescence images of the implants (green: CD29, red: CD34, blue: nuclei). The red triangles pointed out typical CD29^+^CD34^+^ cells. Scale bar = 10 µm. **(B)** Representative anti-CD31 immunohistochemical images of the implants. The red triangles pointed out typical CD31^+^ cells or blood vessels. Scale bar = 50 µm. **(C)** The number of CD29^+^CD34^+^ APs per field of view (scar bar = 10 μm) was analyzed (n = 3). **(D)** The number of CD31^+^ ECs or blood vessels per field of view (scar bar = 50 μm) was analyzed (n = 3). The significance was tested with ordinary one-way ANOVA followed by Tukey’s multiple comparisons test. (*/**/***/**** marked beside the data point represented the comparison between the sEV-AT group/the CL group and the blank group, *p < 0.05, **p < 0.01, ***p < 0.001, ****p < 0.001).

To further investigate the effect of macrophage depletion on the homing of functional cells (APs and ECs), CD29^+^CD34^+^ cells (Figure 4A) and CD31^+^ cells (Figure 4B) were respectively identified as APs and ECs according to previous studies (Rivera-Gonzalez et al., 2016; Shook et al., 2018). Immunofluorescence images displayed there were nearly no positive cells at 3 days and 5 days in each group. At 1 week, the number of CD29^+^CD34^+^ in the sEV-AT group increased significantly, while the other two groups did not show positive cells. At 2 weeks, although APs appeared in the blank group, the number of APs in the sEV-AT group continued to increase and exceeded 6 times that of the blank group. Surprisingly, there were almost no CD29^+^CD34^+^ cells in the CL group from beginning to end, and the number was even lower than that in the blank group (Figures 4A,C).

For ECs and blood vessels, immunohistochemical staining results showed CD31^+^ cells significantly increased in the sEV-AT group compared with the other two groups, in which there were no CD31^+^ cells. 1 week later, immunohistochemical staining showed blood vessels were formed in the sEV-AT group and gradually matured. It was difficult to directly count the number of CD31^+^ cells at the late stage, so the number represented the number of blood vessels. Similar to the infiltration of APs, there were fewer CD31^+^ ECs and blood vessels in the blank group and Cl group compared with that the sEV-AT group (Figures 4B,D).

These results indicated to a great extent the recruitment of APs and ECs by sEV-AT was achieved through macrophages, and the regulatory role of macrophages played a very important role in this process.

### Macrophage depletion eventually led to the failure of adipose tissue regeneration

**FIGURE 5:**
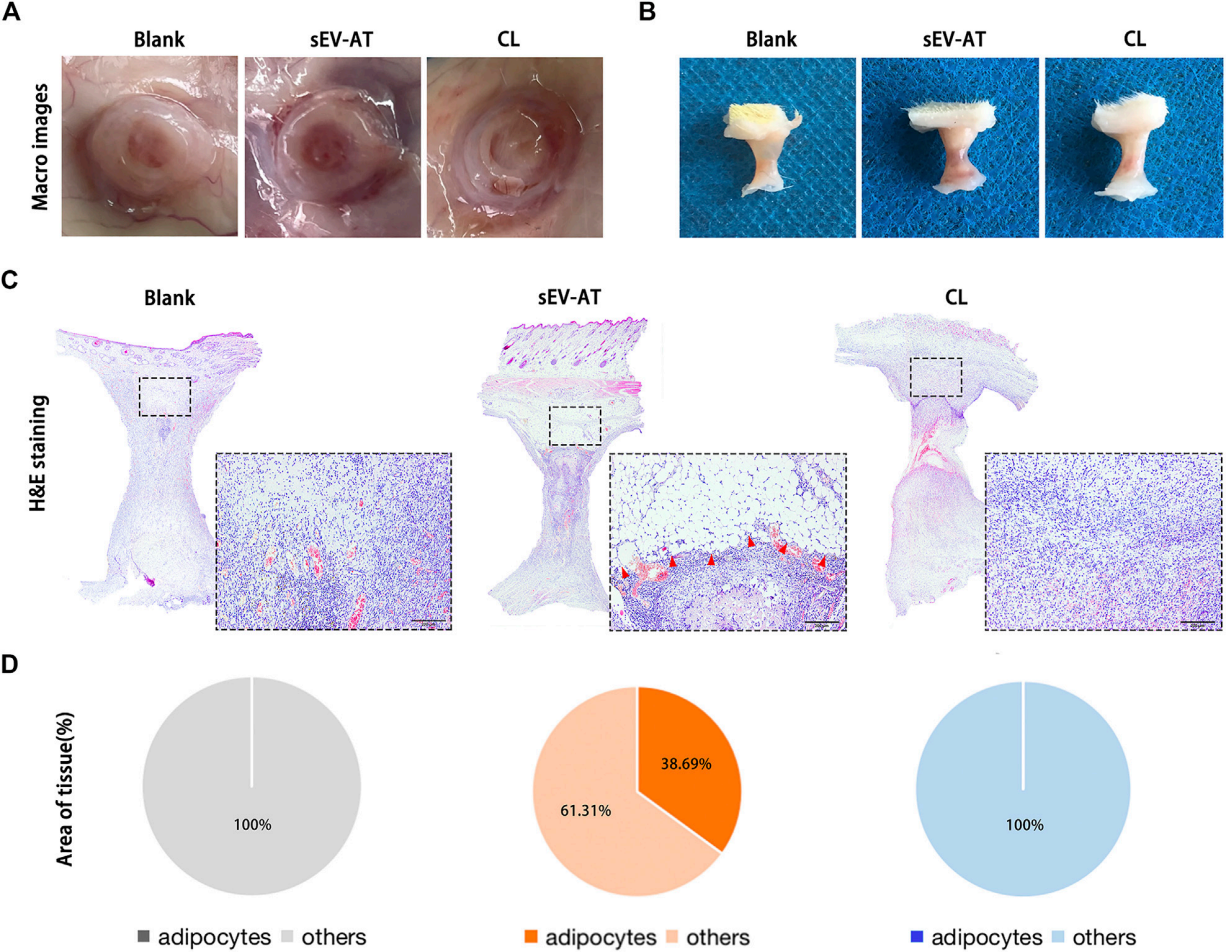
Adipose tissue regeneration in the implants. Representative macro images of the implants **(A)** with the tubes or **(B)** without the tubes at 4 weeks. **(C)** Representative H&E-stained images of the implants at 4 weeks. (Black dotted square: areas were magnified). The red triangles pointed out the regenerated adipose tissue. Scale bar = 200 µm. **(D)** The average percentage of adipose tissue area and other areas in the whole implants (n = 3).

After 4 weeks of implantation, the implants were also used for evaluating adipose tissue regeneration. In general, we observed blood vessels, which extended into the tube, only in the sEV-AT group (Figure 5A). The volume of tissue in these 3 groups showed no difference (Figure 5B). It might be due to the remained Matrigel, which absorbed at 8 weeks according to our previous study. H&E staining showed there were a large number of regenerated adipocytes in the sEV-AT group and the area of adipocytes was about 32% of the whole implants. However, in the CL group, there was no regenerated adipocyte in the implants, which was similar to the blank group (Figures 5C,D).

These results indicated the depletion of macrophages in the early stage of implantation was followed by the decrease of APs and ECs homing, which eventually led to the destruction of adipose tissue regeneration.

### sEV-AT indirectly promoted the migration of APs and ECs *via* modulating macrophages

**FIGURE 6:**
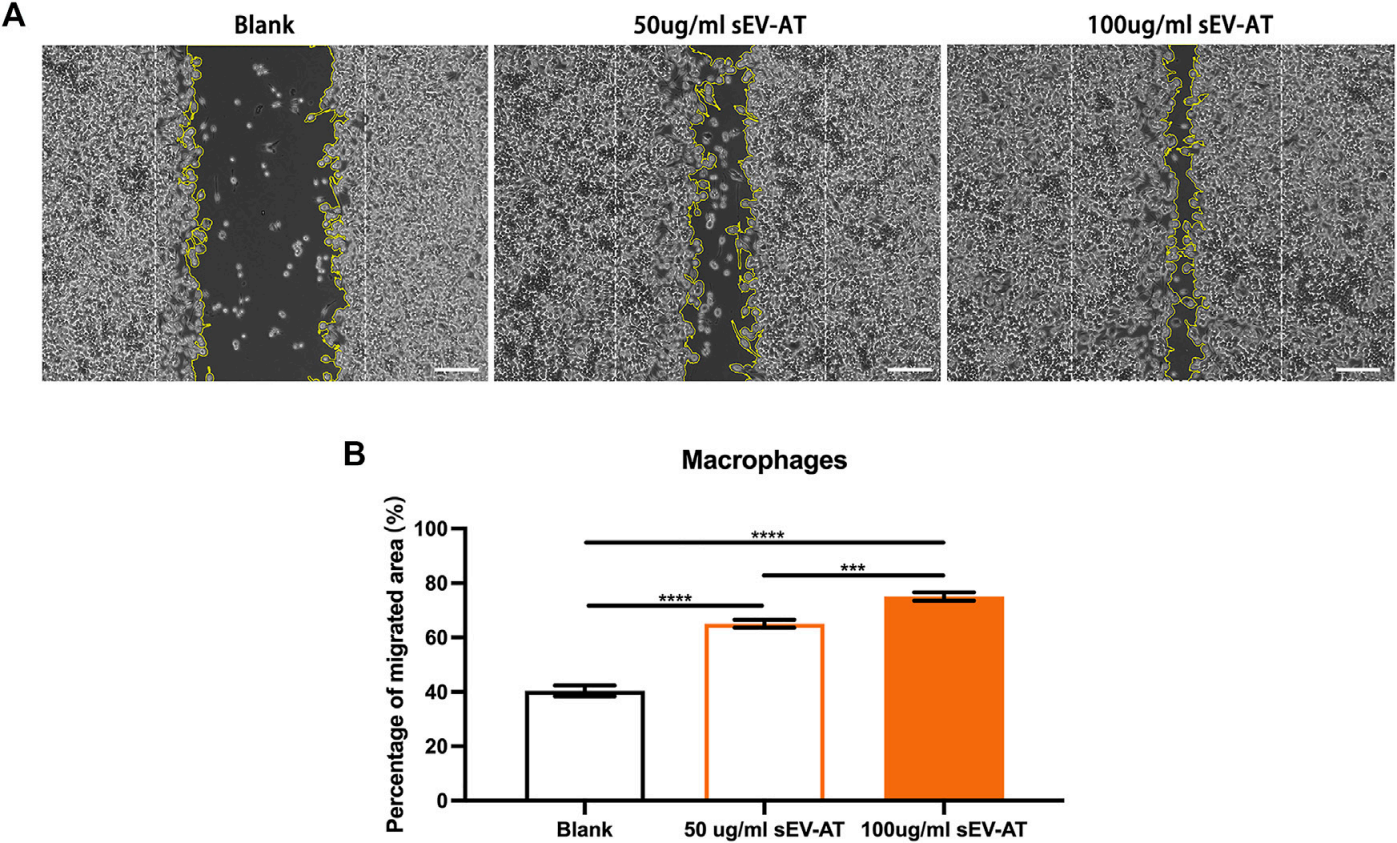
The migration of macrophages induced by sEV-AT *in vitro*. **(A)** Representative images of scratch assays at 30 h. The white dotted lines pointed out the border of the scratches at 0 h. The yellow lines pointed out the border of the migrated cells at 30 h. Scale bar = 200 μm . **(B)** The relative migrated areas per field of view (scale bar = 200 μm) were analyzed (n = 3). The significance was tested with ordinary one-way ANOVA followed by Tukey’s multiple comparisons test. (***p < 0.001, ****p < 0.001).

*In vitro*, we first investigated the effect of sEV-AT on macrophage migration. Macrophages were treated with different doses of sEV-AT (50 and 100 ug/ml) and the results showed within a certain concentration range, sEV-AT could directly promote the migration of macrophages in a dose-dependent manner (Figure 6).

**FIGURE 7:**
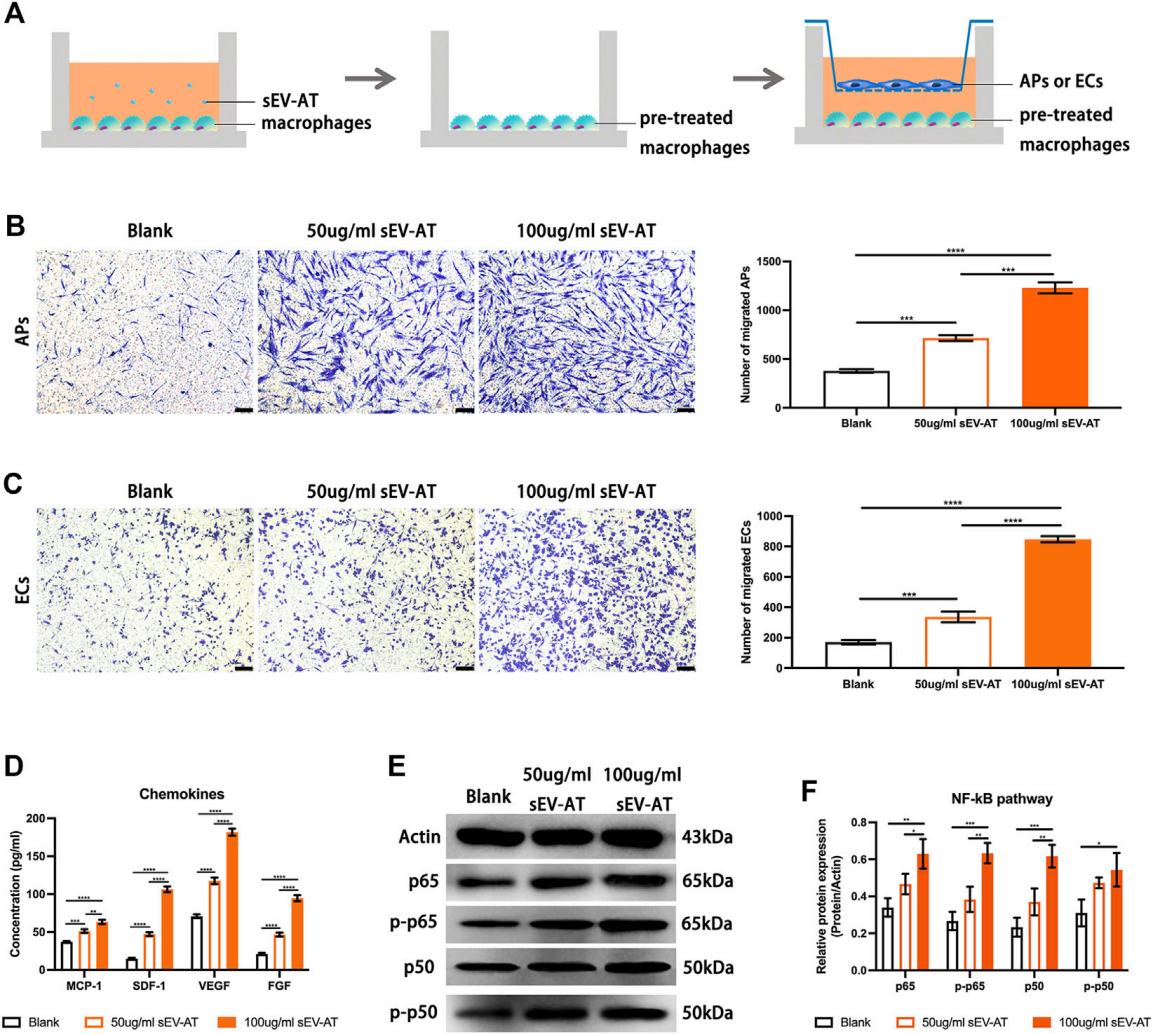
The chemoattraction ability of macrophages induced by sEV-AT *in vitro*. **(A)** Schematic view of the experimental operation process. Macrophages were seeded in the lower layer and cultured with 0, 50, and 100 μg/ml sEV-AT for 6 h, then the culture medium was removed, and APs or ECs were seeded in the upper layer to set up a co-culture system. **(B)** Representative images of migrated APs. Scale bar = 200 µm. Migrated APs per field of view (scale bar = 200 µm) were analyzed (n = 3). **(C)** Representative images of migrated ECs. Scale bar = 200 µm. Migrated ECs per field of view (scale bar = 200 µm) were analyzed (n = 3). **(D)** The concentration of chemokines (SDF-1, MCP-1, VEGF, and FGF) in the culture medium was analyzed (n = 3) by ELISA. **(E)** Western blot and **(F)** semi-quantification of the expression of NF-kB signaling pathway-related proteins (p50, p65, phosphorylated-p50, and phosphorylated-p65) in macrophages induced by sEV-AT (n = 3). The significance was tested with ordinary one-way ANOVA followed by Tukey’s multiple comparisons test. (**p < 0.01, ***p < 0.001, ****p < 0.001).

To further investigate the ability and mechanism of the homing of APs and ECs induced by sEV-AT-pretreated macrophages, we performed a co-culture system. We firstly treated macrophages with 50 and 100 ug/ml sEV-AT for 6 h and co-cultured these pretreated cells with APs or ECs (Figure 7A). The results showed sEV-AT-pretreated macrophages could both promote the migration of APs and ECs in a dose-dependent manner (Figures 7B,C). Then, chemokines (SDF-1, MCP-1, VEGF, and FGF), reported to promote the migration of APs or ECs preciously, were selected to be evaluated by ELISA. The results indicated the concentrations of SDF-1, MCP-1, VEGF, and FGF in the medium were increased by sEV-AT treatment (Figure 7D). In addition, western blot analysis showed the expression of NF-kB signaling pathway-related proteins (p50, p65, phosphorylated-p50, and phosphorylated-p65) increased in a dose-dependent manner after sEV-AT treatment (Figures 7E,F).

These results indicated sEV-AT could not only increase the secretion of chemokines by recruiting more macrophages, but also increase the secretion of chemokines by activating NF-kB signaling pathway in macrophages.

## Discussion

It was becoming increasingly appreciated the importance of modulating and understanding the inflammatory response in regenerative medicine. After implanting the biomaterial into the body, the implantation inevitably evoked a cascade of immune responses, which began with inflammation and was followed by cell proliferation and tissue regeneration (Anderson and McNally, 2011). Previous studies demonstrated macrophages played diverse functions, including scavenging debris (Peng et al., 2007; Wynn and Vannella, 2016), recruiting host cells (Wang et al., 2018), promoting vascularization (Sun et al., 2017), and inducing cell differentiation (Wang et al., 2017). Most previous studies focused on the anti-inflammation ability of sEVs to polarize M1 macrophages to M2 macrophages (Kwak et al., 2022; Li et al., 2022; Liu et al., 2022; Tang et al., 2022; Yang et al., 2022; Zhao et al., 2022). However, the role of macrophages in cell recruitment was largely unknown, especially in the process of tissue regeneration induced by sEVs.

In this study, compared with the blank group, a significant increase in infiltration of F4/80^+^ macrophages would be detected within 1 week in the sEV-AT group. *In vitro* results also showed sEV-AT could significantly promote the migration of macrophages. Then, to verify whether macrophages played an indispensable role in the process of adipose tissue regeneration, we used clodronate liposome to deplete macrophages in the CL group. Clodronate liposome was a kind of reagent commonly used for depleting monocytes/macrophages *in vivo* or *in vitro* (Li et al., 2016; Wang and Kubes, 2016). According to the instructions, most of the active ingredient disodium, clodronate, would be excreted with urine within about 48 h after injection, so we injected clodronate liposome every 2 days to ensure that macrophages were continuously depleted in the early stage of implantation. Nearly no F4/80^+^ macrophages infiltrated the implants in the CL group, which indicated clodronate liposome injection could not only deplete the macrophages in the livers and spleens but also showed an excellent depletion effect on the macrophages around the implants. Taken together, these results directly suggested a rapid and increase infiltration of macrophages could be induced by sEV-AT and clodronate liposome injection was effective enough to prevent the early infiltration of macrophages.

In the process of adipose tissue regeneration, the recruitment and differentiation of functional cells (adipocyte precursors and endothelial cells) was important. To further investigate the relationship between macrophages and functional cells, *In vivo*, we found the rapid infiltration of macrophages was followed by the increased homing of APs and ECs in the sEV-AT group. However, the infiltration of functional cells was also damaged by the depletion of macrophages. *In vitro*, SDF-1, MCP-1, VEGF, and FGF in the co-culture system were significantly increased by sEV-AT, which were generally believed to promote the homing of APs and promote angiogenesis (Stuermer et al., 2015; Zhao and Zhang, 2016). Taken together, these results suggested macrophages played a connecting role in the infiltration of APs and ECs induced by sEV-AT and a lot of chemokines might be involved in this process.

The nuclear factor-kappa B (NF-kB), as a key transcription factor, modulated cell survival, cell proliferation, and immune response (Liu et al., 2017; Sun, 2017). The activation of NF-kB was related to various inflammatory diseases, such as autoimmune diseases, metabolic disorders, and cancers (Baker et al., 2011). Excessive activation of NF-kB in macrophages would lead to inflammatory diseases *via* the increase of proinflammatory mediators (Wang et al., 2014; Liu et al., 2017). However, recent studies have shown that the activation of NF-kB signaling pathway was also related to the secretion of many chemokines. For example, X. Song found that activating NF-kB signaling pathway increased the expression of CCL2 and CXCL10 in macrophages (Song et al., 2021). Hong et al. (2021) found the LPS-activated exosomes enhanced the expression of CCL4, CCL17, and CCL19 in macrophages *via* activating the MyD88/NF-κB signaling pathway. In this study, we preliminarily explored the increase of chemokines induced by sEV-AT might be related to the activation of NF-kB signaling pathway. We were not sure if it was the only pathway or even the most important pathway. However, this provided a possible direction for further deep research.

**FIGURE 8:**
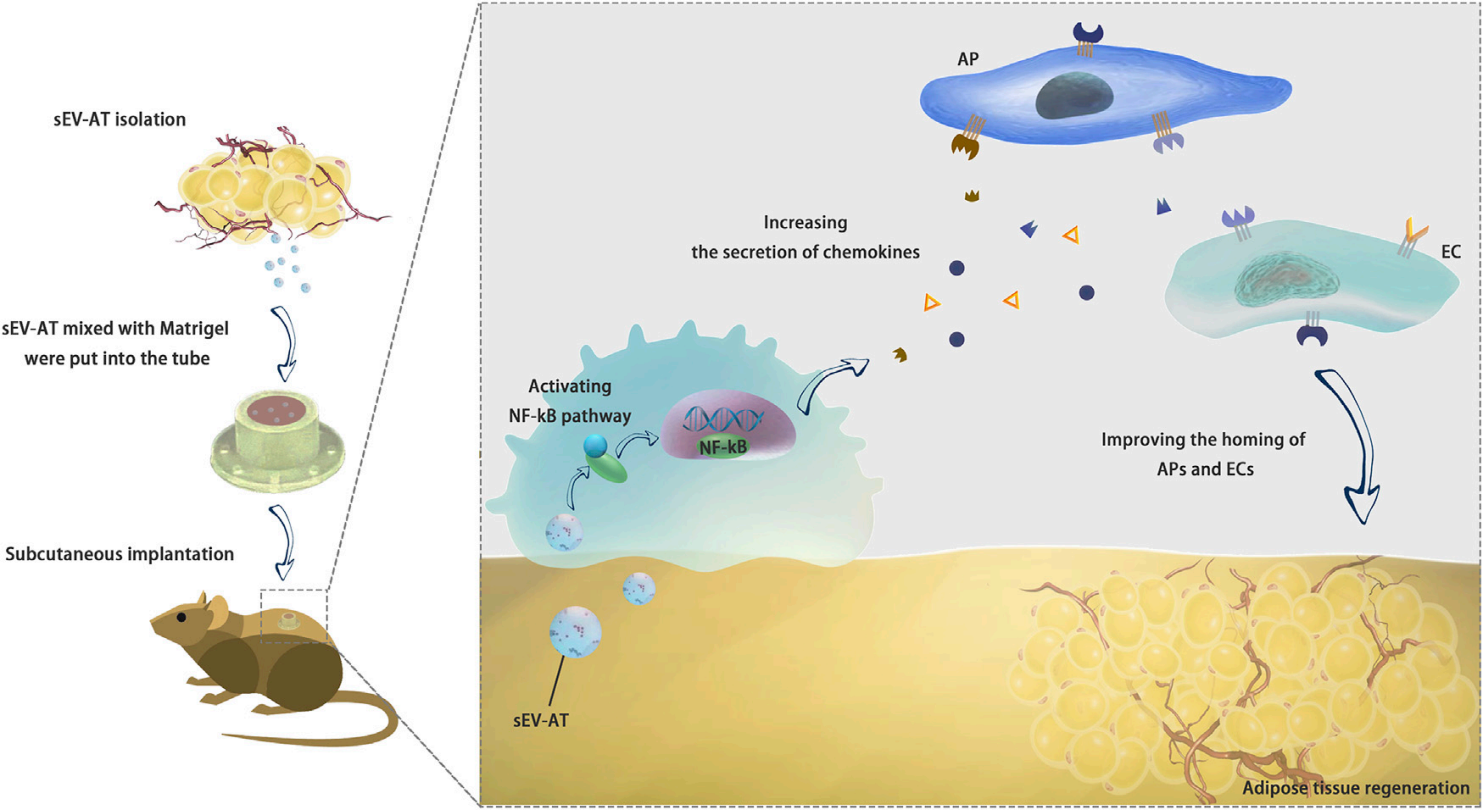
Conclusion. sEV-AT might regulate the secretion of chemokines *via* activating NF-kB signaling pathway in macrophages to improve the homing of APs and ECs and facilitate adipose tissue regeneration.

In the past few years, we found sEV-AT could effectively induce adipose tissue regeneration (Dai et al., 2017) and adipose tissue extract (ATE) also showed the same ability (He et al., 2022). We also found the ability of sEV-AT derived from rats and porcine to repair soft tissue was similar. We have been trying to explain why sEV-AT could promote adipose tissue regeneration. Previously, we tested the ability of sEV-AT to induce the adipogenic differentiation of APs and the angiogenesis of ECs (Dai et al., 2017; Zhang et al., 2017; Dong et al., 2020). However, how host cells were recruited into implants was unknown. This study suggested sEV-AT might promote the release of chemokines in macrophages *via* activating NF-kB signaling pathway. SDF-1 and MCP-1 might play a chemotactic role in AP recruitment. VEGF and FGF contributed to the homing of ECs and blood vessel formation (Figure 8).

Despite these encouraging results, there were some limitations. For example, how sEV-AT promoted macrophage recruitment into implants and which components of sEV-AT participated in macrophage regulation were unknown. Besides, the phenotype of macrophages contributed to adipose tissue regeneration has not been studied. Hence, we will further study and gradually answer these questions.

## Data Availability

The original contributions presented in the study are included in the article/Supplementary Material, further inquiries can be directed to the corresponding author.
